# Age-Specific Seroprevalence of Hepatitis A Virus in Turkey Between 2000 and 2023: Systematic Review and Meta-Analysis

**DOI:** 10.3390/diagnostics14222464

**Published:** 2024-11-05

**Authors:** Ihsan Hakki Ciftci, Mehmet Koroglu, Tayfur Demiray, Huseyin Agah Terzi, Elmas Pinar Kahraman Kilbas

**Affiliations:** 1 Department of Medical Microbiology, Faculty of Medicine, Sakarya University, Sakarya 54100, Turkey; 2 Department of Medical Laboratory Techniques, Health Services Vocational School, Fenerbahce University, Istanbul 34758, Turkey; elmspnrkk@gmail.com

**Keywords:** hepatitis A virus, meta-analysis, seroprevalence, Turkey

## Abstract

**Background**: Hepatitis A virus (HAV) is a leading cause of acute viral hepatitis and is primarily transmitted by the fecal–oral route. The clinical presentation and progression of the disease varies according to the age of the patient. Turkey is classified as a moderately endemic country, and HAV infection continues to be an important public health problem worldwide. **Methods**: In this study, a systematic meta-analysis was conducted to evaluate age-specific HAV seroprevalence rates in Turkey between 2000 and 2023. A comprehensive literature review identified 57 articles that met the inclusion criteria. The studies were assessed for quality, and seroprevalence rates were evaluated across four different age groups. Statistical analyses were performed using Comprehensive Meta-Analysis (CMA) software (CMAVersion 3.0) and SPSS (SPSS Statistics 25.0). **Results**: HAV seroprevalence rates were found to be 73.18% in the 0 < 5 age group and 90.90% in the >35 age group. The overall seroprevalence estimated using a random effects model was 64.5% (95% CI: 58.3–70). High heterogeneity was observed among the studies, and the prevalence estimates changed when low-quality studies were excluded. **Conclusions**: This meta-analysis suggests that the increasing trend in HAV IgG seroprevalence in Turkey, especially among young populations, is likely due to the vaccination program initiated in 2012. Furthermore, the heterogeneity observed among regions highlights the importance of regional public health strategies. Future studies should focus on providing more detailed data to evaluate the long-term effects of vaccination and to explain regional differences in HAV seroprevalence.

## 1. Introduction

Hepatitis A virus (HAV) is one of the main causes of acute viral hepatitis and is easily transmitted via the fecal–oral route [1,2,3]. The clinical presentation and course of the disease varies according to the age of the patient. Hepatitis A virus infection is usually asymptomatic in young children. Jaundice, fatigue, nausea and other symptoms become more common with age. In very rare cases, HAV can cause liver failure and death. The incidence of HAV is associated with basic socioeconomic factors, such as per capita income, sewage infrastructure, hygiene and access to clean water [2,3,4]. The seroprevalence of HAV infection is decreasing worldwide, except in underdeveloped and some developing countries; however, HAV infection remains a significant public health problem [5].

The World Health Organization (WHO) divides geographical regions into high, medium and low endemic areas for HAV infection [6,7]. In high endemic areas with poor hygiene conditions, children come into contact with HAV before the age of ten and develop lifelong immunity. Symptomatic HAV infections and HAV outbreaks are rare in such countries [3]. In moderately endemic areas, contact with the virus usually occurs later in life, and acute hepatitis with HAV usually affects adolescents and young adults. As countries and sanitation conditions improve, the age at which HAV infection is acquired increases as the incidence rate decreases [3]. Hepatitis A virus outbreaks can occur in low-endemic areas, and the course of the disease becomes more severe and complex with increasing age [2,4,5]. Age is an important factor in predicting the prognosis and outcome of the disease due to changing clinical patterns associated with age. The mean age of infection increases due to the decreasing endemicity rate, thereby increasing the burden on health systems. Turkey is reported as a moderately endemic country in global reports [6,7,8]. Considering the variability of socioeconomic levels and sanitation conditions in different geographical regions, it is interesting to examine how age-specific seroprevalence changes over the years.

There is only one serotype of hepatitis A virus, so after recovery from the disease, lifelong immunity is established with IgG-type antibodies [9]. Infection rates in a population can be monitored with IgG anti-HAV and interpretations can be made about HAV seroprevalence [3]. Age-specific categorization of HAV seroprevalence provides an indirect measure of age-specific incidence rates of HAV infections and is considered the best way to describe the status of HAV infection in a country [10]. Age-specific HAV seroprevalence is also an important parameter in determining the appropriate age for vaccination and routine immunization [5,11]. However, incidence studies based on case reports are subject to data deficiencies and bias. Although many studies in Turkey have investigated HAV seroprevalence rates in various provinces, there is a lack of comprehensive assessment of age-specific trends at the national level. This study aimed to fill this gap through a meta-analysis of seroprevalence data from the last 23 years.

## 2. Materials and Methods

### 2.1. Systematic Literature Search

**Figure 1:**
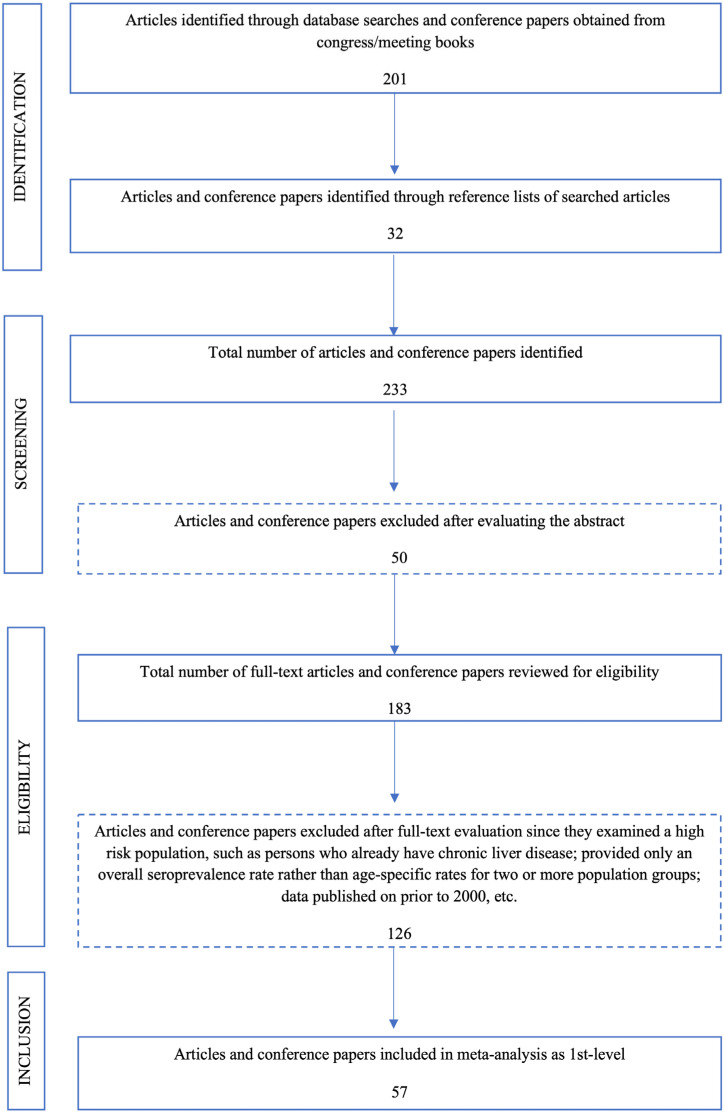
Flowchart scheme for the systematic literature search and selection of studies.

A systematic search was performed in PubMed, Google Scholar and the online database of the Library of Sakarya University (which searches Web of Science, EBSCOhost, SCOPUS, ULAKBIM Medical database, Turkmedline, etc.) both in Turkish and English languages to identify published articles, conference abstracts and other online resources. We used a systematic search algorithm similar to previously proposed search algorithms (Figure 1) [1,12]. The search was performed using the following keywords: “hepatitis A”, “HAV”, “hepatitis A seroprevalence in Turkey”, “HAV seroprevalence in Turkey”, “hepatitis A prevalence in Turkey”, “HAV prevalence in Turkey”. We reviewed the reference lists of all articles and collected all published data between 2000 and 2023. This period was chosen to evaluate seroprevalence trends before and after the national HAV vaccination program launched in Turkey in 2012.

### 2.2. Categorical Scoring and Extraction of the Data

All the eligible studies were given a quality score according to previously determined criteria [1,13,14]. The basic criteria for categorical scoring include the sampling method, age group ranges, study population and diagnostic laboratory method.

1. Sampling method: equally and equiponderant samples were selected from urban and rural areas (10), randomly chosen from a specific group (8), simply and randomly selected (5), non-randomized (1) and not identified (0).
2. Age group ranges: all ages (0–80) were included (10), 80–100% of all ages (8), 60–80% (6), 40–60% (4), 20–40% (2) and less than 20% (1).
3. Study population: whole country (5), whole region or province (4), districts or neighborhoods (3) and only one district/neighborhood (1).
4. Diagnostic laboratory method: Chemiluminescence, Enzyme-Linked Immunosorbent Assay (ELISA), Enzyme-Linked Fluorescent Assay (ELFA), other methods (2), immunochromatic tests (card tests) (1) and not identified (0).

After scoring with the given chart, the mean value and standard deviation (SD) were calculated. Studies with an SD of +1 and higher were assigned high quality, whereas those with an SD-1 and lower were assigned low quality. Papers lying in between SD ± 1 were accepted as medium quality.

### 2.3. Inclusion Criteria

The inclusion criteria were as follows:

- Studies published in or after 2000;
- Studies containing age-specific seroprevalence data in at least one age group;
- Only original articles that do not focus on high-risk groups, such as healthcare workers, military personnel, sewer workers, etc., are included.

### 2.4. Exclusion Criteria

The exclusion criteria were as follows:

- Review articles, epidemic research, animal studies and environmental research;
- Studies involving individuals vaccinated with HAV or investigating the efficacy of vaccination, genetics and other laboratory-based studies;
- Studies evaluating only IgM anti-HAV;
- Studies examining particular patient groups, such as dialysis patients, those with acute or chronic liver disease, liver transplant patients and patients with other chronic infections;
- Reports on overall seroprevalence rates and studies on HAV infection incidence rates;
- Studies with general seroprevalence rates instead of age-specific values;
- Multicenter studies and studies with inconsistent data were not included in this study.

Duplicate studies that appeared in more than one database were removed. The systematic review and meta-analysis were conducted using the PRISMA guidelines (Figure 1) [15,16].

### 2.5. Age Groups

We found that researchers used various age groupings in different studies, which indicated a lack of standardization for determining the age groups. We divided ages into four different age groups to analyze and compare the age-specific data more appropriately and accurately. The age groups were classified as 0 < 5 years, 5 < 15, 15–35 and >35 years. We also preferred not to use overall rates of HAV seroprevalence. The WHO reports were taken into consideration when determining the age groups [17].

### 2.6. Statistical Analysis

We calculated pooled estimates with 95% confidence intervals (CIs) both within age groups and overall across all studies, using both fixed and random effects models, with Comprehensive Meta-Analysis (CMA) ver. 3.0 software (Biostat, Englewood, CO, USA). Homogeneity across studies was assessed using the I squared statistic. In the presence of significant heterogeneity (I^2^ > 50%), Cochrane’s Q test was applied. A level of <0.01 was considered as significant.

Descriptive statistical analyses (mean, SD, etc.), normality distribution, and analysis of differences between groups (One-Way Anova test) were calculated using SPSS software (IBM SPSS Statistics, Version 25.0; IBM Corp., Armonk, NY, USA). Parametric tests were used for data conforming to a normal distribution. A *p*-value of less than 0.05 was considered significant.

## 3. Results

After a systematic literature search, as described above, we reviewed 195 articles and six conference proceedings with age-specific HAV seroprevalence data. The database search using keywords yielded 201 studies. In addition, the search of the bibliographies of these articles yielded 32 publications. After examining the abstracts, 50 irrelevant studies and 126 additional studies that did not meet the inclusion criteria were eliminated. A total of 57 original articles were included in this meta-analysis (Figure 1) [18,19,20,21,22,23,24,25,26,27,28,29,30,31,32,33,34,35,36,37,38,39,40,41,42,43,44,45,46,47,48,49,50,51,52,53,54,55,56,57,58,59,60,61,62,63,64,65,66,67,68,69,70,71,72,73,74].

In the 0–<5 age group, the highest HAV prevalence rates were calculated as 44.44%, 25.57% and 23.43% between 2015 and 2023, 2008 and 2014 and 2001 and 2007, respectively. In the 5–<15 age group, the highest HAV prevalence rates were calculated as 51.90%, 46.89% and 31.81% between 2001 and 2007, 2015 and 2023 and 2008 and 2014, respectively. In the 15–35 age group, the highest prevalence rates were calculated as 64.69%, 61.97% and 47% between 2001 and 2007, 2008 and 2014 and 2015 and 2023, respectively. The highest prevalence rates in the age group of >35 years and above were found to be 96.23%, 94.19% and 90.90% between 2008 and 2014, 2001 and 2007 and 2015 and 2023, respectively.

**Figure 2:**
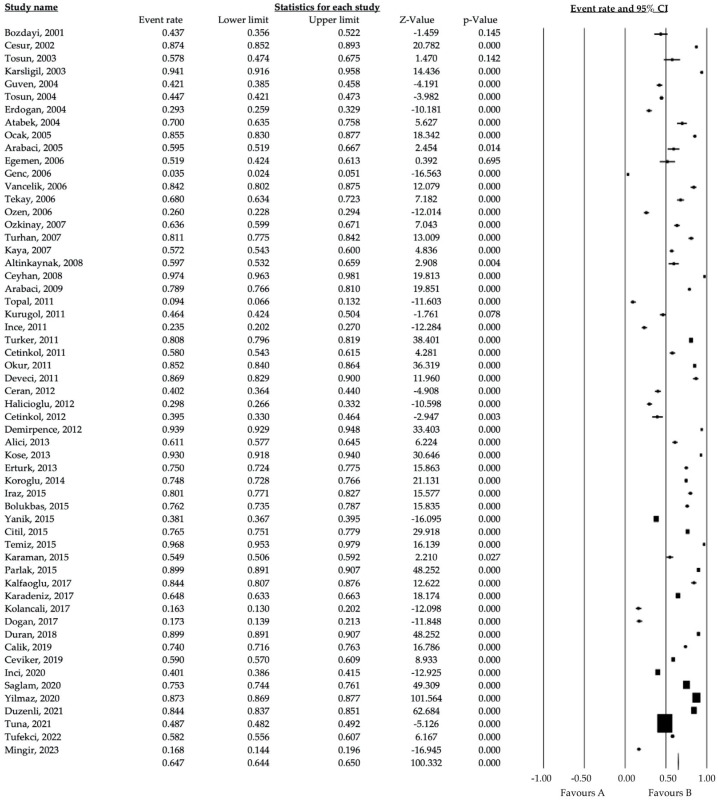
Forest plot analysis of the age-specific HAV seroprevalence studies in Turkey [18,19,20,21,22,23,24,25,26,27,28,29,30,31,32,33,34,35,36,37,38,39,40,41,42,43,44,45,46,47,48,49,50,51,52,53,54,55,56,57,58,59,60,61,62,63,64,65,66,67,68,69,70,71,72,73,74].

We applied a “forest plot analysis” to eligible HAV seroprevalence studies (Figure 2). We presented the results of both fixed and random effects models. However, including comments from the random effects model in this analysis may be appropriate since the age-specific HAV seroprevalence studies were heterogeneous.

In this meta-analysis, we evaluated 57 published articles on age-specific HAV seroprevalence, including a total of 157,836 patients. Pooling the results extracted from all included reports, independent of study design, yielded an event estimation of 64.5% for the random effects model (95% CI: 58.3–70.2) and 63.7% (95% CI: 63.5–64) for the fixed effects model (Q^2^ = 25085.65, df = 57, *p* < 0.0001, I^2^ = 99.77). In the comprehensive meta-analysis, event rates calculated with fixed and random effects models yielded similar rates and consequently showed high sensitivity (Table 1).

According to categorical scoring, the studies ranged between 7 and 22. The number of included articles according to high, medium and low category scores was 9, 42 and 6, respectively. After removing low-quality articles, the point estimate of HAV prevalence in the random effects model shifted from 61.8% (95% CI: 60.5–63.2). The negative effect of articles with low-quality scores on seropositivity rates was limited.

**Figure 3:**
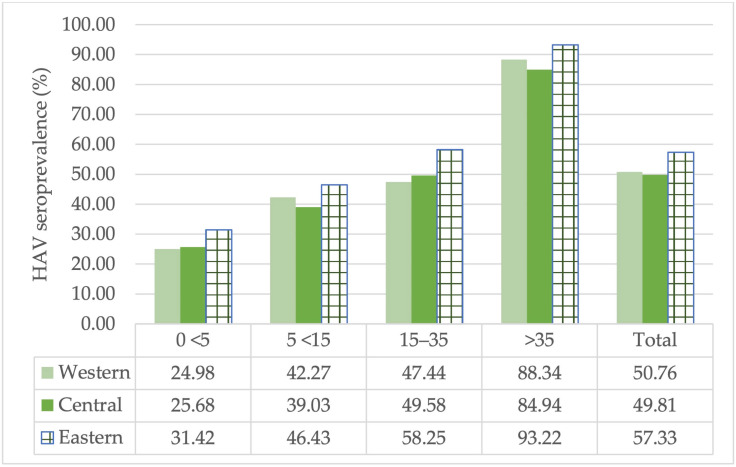
The HAV seroprevalence rates by region in Turkey.

The seroprevalence rate was found to be lower in the central and western regions than in the eastern region across all age groups. A statistically significant difference was found between the seroprevalence rates of the 0–<5 and 15–35 age groups and geographical regions (*p* < 0.05). Additionally, the articles included in this study from the eastern region contain high and medium endemicity notifications. It may be approaching the intermediate endemicity rates of the eastern region, but the lack of data for subsequent years in 2020 does not provide an accurate assessment. As a result, the data presenting appropriate information on age groups in the evaluated studies indicate that the incidence rate remains higher in the eastern region of the country than in Turkey’s other regions (Figure 3).

**Figure 4:**
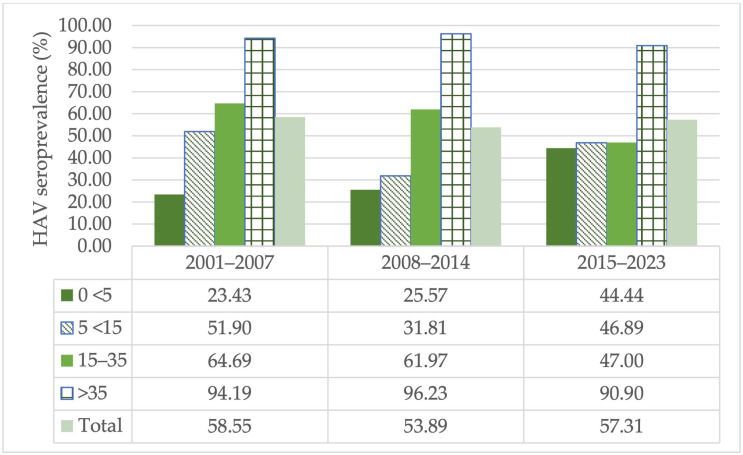
The HAV seroprevalence rates by age group over the years in Turkey.

The seroprevalence rate was calculated for the 2001–2007 (58.55%), 2008–2014 (53.89%) and 2015–2023 (57.31%) year groups. A statistically significant increase was detected in the 0–<5 age group over the years (*p* < 0.05). Compared to 2001–2007, a statistically insignificant decrease in HAV seroprevalence was observed in both the 2008–2014 and 2015–2023 periods for the 5–<15 and 15–35 age groups. A minimal decrease in HAV seroprevalence was observed for the >35-year age group over the 23 years examined in this study. As a result, the studies presenting appropriate data on age groups show that HAV seroprevalence has decreased over the years in all age groups except for the 0–<5 age group (Figure 4).

HAV IgG analyses were performed using Enzyme-Linked ImmunoSorbent Assays (ELISAs) in thirty-six of the included studies, Chemiluminescence/Electro-Chemiluminescence Immunoassays (CLIAs/ECLIAs) in eight studies, Chemiluminescent Microparticle Immunoassays (CMIAs) in eight studies, Microparticular Enzyme Immunoassays (MPEIAs) in two studies, Enzyme-Linked Fluorescent Assays (ELFAs) in one study, and the study method was not reported in two studies. No statistically significant difference was found between the method used and HAV IgG seroprevalence (*p* > 0.05).

## 4. Discussion

From 1990 to 2021, an increase of approximately 13.9% in the incidence of HAV has been reported worldwide [75]. A meta-analysis conducted in 2024 found a 32% cumulative hospitalization rate among HAV-infected patients despite increased vaccination rates [76]. This increases the potential burden of HAV outbreaks on the existing healthcare system. Therefore, widespread screening of adults in at-risk groups for HAV and the reporting of these results are important.

Although it is known that the most effective ways to prevent the spread of HAV infections are improved sanitation, food safety and vaccination, necessary actions have not yet been taken worldwide. This meta-analysis was conducted to promote a better understanding of HAV epidemiology by bringing together individual studies examining HAV seroprevalence rates and diagnostic methods in Turkey, emphasizing the importance of screening tests in high-prevalence subgroups and the need for preventive measures.

Over the last 20–30 years, countries have been classified as high, moderate, low and very low endemic based on the age of exposure and anti-HAV IgG seropositivity. A high endemic region is defined as having 90% or above immunity up to the age of 10; a moderate endemic region is defined as having 50% or above immunity up to the age of 15 and less than 90% immunity up to the age of 10; a low endemic region is defined as having more than 50% immunity up to the age of 30 and less than 50% immunity up to the age of 15; and a very low endemic region is defined as having 50% or above immunity up to the age of 30 [2]. In studies conducted before 2010, Turkey was described as moderately endemic [24]. When the endemicity levels of the studies included in this meta-analysis were examined, it was seen that 17 were high, 6 were very high, 28 were moderate and 6 were low endemic. The endemicity level in Turkey was detected at a low level in all periods covering the years 2001–2007, 2008–2014 and 2015–2023. However, it was determined that the HAV IgG seropositivity rate for the age group 0–<5 years increased during the period 2015–2023. Additionally, the low HAV seroprevalence rates in the 0–<5, 5–<15 and 15–35 age groups between 2008–2014 may be due to the fact that most studies were conducted in the western and central Anatolian regions.

The HAV vaccination program was included in the Childhood Vaccination Program (CVP) at the end of 2012 and is administered to children born after March 2011 in two doses at 18 and 24 months in Turkey [77]. Unfortunately, current scientific publications in Turkey are insufficient to demonstrate the effects of the CVP on HAV immunity. Only two studies reporting seropositivity and vaccination information for the 0–<5 age group reported anti-HAV positivity above 90%. The insufficient sample sizes in these two studies, with relative weights of 0.49 and 1.95, do not allow for an evaluation of the future perspective of the CVP. However, in some studies conducted in our country, HAV seroprevalence in children aged 0–5 years was reported as 25.8% and 27.35% in 2004 and 2007, respectively, and 54.23% and 58.87% in 2018 and 2020, respectively [25,35,65,70]. In the studies included in this meta-analysis, it was observed that the average seroprevalence rate in the 0–<5 age group increased from 25.16% before 2012 to 40.99% after 2012. This situation was attributed to the implementation of the CVP in our country and is considered an expected finding.

When we excluded low-quality articles, we found a minimal decrease in the prevalence rates in this meta-analysis. In order to prevent misinterpretations in the future, studies should be carefully planned with a more comprehensive and representative sample population reflecting the entire country, covering all age groups and taking into account the CVP.

Regional differences have been reported in Brazil, Mexico, Iran and Italy [1,78,79,80]. Heterogeneous seroprevalence rates were observed in different regions of Turkey (Figure 3). The western part of our country shows a lower seroprevalence rate due to its more developed socioeconomic status. Except for the 0–<5 age group, seroprevalence rates gradually decreased in the central and western regions. In all age groups, the seroprevalence rate in the eastern region was higher than in the other regions. However, only the seroprevalence rates of the 0–<5 and 15–35 age groups were found to be statistically significantly higher in the eastern region compared to the western and central regions. Factors contributing to the high seroprevalence rate in the eastern regions include crowded households, population growth, the settlement of immigrants in these regions since the influx of immigrants from Syria to Turkey in 2011 and the existence of refugee camps. Additionally, since there was insufficient data after 2020, we have not been able to comment on the latest status of seropositivity rates in the eastern regions. Standardization of screening and confirmatory tests may be recommended to minimize regional differences. However, no significant difference was found between the method used in our study and seroprevalence. Therefore, screening tests and immunization should be conducted in regions with low socioeconomic status and should be given the same attention as in the least developed regions.

It is known that more than 90% of children living in low socioeconomic regions and more than 90% of young adults in developing countries have HAV seropositivity [81]. In our study, regardless of regional differences, from 2001 to 2023, HAV seropositivity in the 5–<15, 15–35 and >35 age groups decreased gradually by 5.0%, 17.7% and 3.29%, respectively. When the CVP is ignored, this situation can be evaluated as an improvement in drinking water systems and sanitation conditions in Turkey. However, any lapse in hygiene may expose the population to an epidemic in regions with high immigration. Therefore, basic lifestyle information, such as socioeconomic status, access to clean water resources and living area (rural or urban), must be included in newly planned studies.

In seroprevalence studies, it is a matter of concern whether the selected sample represents the general prevalence. Therefore, the exclusion criteria we used should be taken into account when interpreting the results. Our study excluded groups with a high prevalence of HAV infection (such as healthcare workers, chronic hepatitis patients, students living in dormitories, prisoners and military personnel), known vaccinated samples and studies that included data from immunosuppressed patients. Despite these exclusion criteria and the total sample size, selection bias cannot be completely excluded.

Despite the 12 years that have passed, information on HAV immunity obtained through vaccination cannot be presented due to the insufficient or incomplete data in the publications that meet the inclusion criteria for this study. Vaccination should be taken into account in new studies to reveal the effects of the CVP. Otherwise, it will not be possible to assess or interpret the future implications of HAV vaccination.

It has been suggested that antibodies against viral hepatitis viruses may have protective roles against coronavirus infection [82,83]. In related studies, the protective role of HAV antibodies and immune system stimulation in patients has been described [82]. During the COVID-19 pandemic, a milder increase in the number of cases and a lower mortality rate compared to developed countries was reported in underdeveloped countries [84,85]. This situation can be explained by the cross-protection of existing immunity against viruses such as HAV, which are endemic in underdeveloped areas. However, there are also studies reporting that there is no significant relationship between the two [86]. The ongoing anti-vaccine sentiment during the COVID-19 pandemic may have affected HAV prevalence, which decreased between 2015 and 2023, especially after the age of 15.

### Strengths and Limitations

This study is one of the few analyses that brings together HAV prevalence and serological diagnostic methods worldwide and was conducted for the first time in Turkey. There was high heterogeneity among the reports from different regions. Heterogeneity was investigated through subgroup analysis. The medical characteristics of the patients, such as chronic diseases and vaccination status, may have contributed to this heterogeneity. Although subgroup analyses were conducted, the aforementioned factors could not be taken into account due to the lack of data in the studies. In addition, the age ranges in the studies reporting seroprevalence for age groups were quite diverse. Therefore, data that did not fit the age groups we determined were excluded when performing statistical analyses.

## 5. Conclusions

A systematic meta-analysis of the age-specific seroprevalence rates of HAV in Turkey over the last 23 years provides important data on the changing epidemiology of the virus. Hepatitis A virus seroprevalence has increased significantly in the 0–<5 age group, associated with the launch of the HAV vaccination program in 2012. Although overall prevalence remains concerning, especially in certain age groups and regions, trends suggest a gradual shift towards lower endemicity.

The heterogeneity observed across regions highlights the impact of socioeconomic factors and access to sanitation on HAV transmission dynamics. Further HAV seroprevalence studies should be planned to include sociodemographic data, such as patient gender, age group, access to clean water, vaccination status, ethnicity, occupation, income level, living area, region of residence, as well as the diagnostic method used and sensitivity and specificity values calculated when the relevant method is compared with the reference method. It is vital that studies adopt standardized methodologies and include a comprehensive demographic representation to better assess the effectiveness of public health interventions. Ensuring homogeneity of the data will contribute to epidemiological research. In addition, increasing the number and quality of seroprevalence studies after 2012, when the HAV vaccination program began in Turkey, will facilitate discussions on the effectiveness and importance of the vaccine.

Continuous monitoring and evaluation of the impact of the vaccination program on seroprevalence will be important in guiding public health strategies and achieving long-term control of HAV in Turkey. As we move forward, prioritizing high-quality research and addressing regional disparities will be vital in our efforts to combat HAV and protect public health.

## Figures and Tables

**Table 1:** Event rate values according to age-specific HAV seroprevalence studies in Turkey.

Age Groups	Event Rates for Fixed and Random Effects Models (95% CI)			
	Fixed (%) (95% CI)	Random (%) (95% CI)	I^2^	Q^2^
0 < 5	49.1 (47.3–50.9)	35.4 (23.8–48.9)	97.88	849.22
5 < 15	54.6 (53.4–55.7)	47.3 (37.5–57.3)	98.39	1620.64
15–35	66.1 (65.6–66.6)	70.7 (62.9–77.5)	99.48	5784.07
>35	69.2 (68.5–69.8)	95.8 (93–97.5)	99.50	5241.76
Total	63.7 (63.5–64)	64.5 (58.3–70.2)	99.77	25,085.65

## Data Availability

The authors declare that all related data are available from the corresponding author upon reasonable request.
